# Complex urban environments provide *Apis mellifera* with a richer plant forage than suburban and more rural landscapes

**DOI:** 10.1002/ece3.9490

**Published:** 2022-11-08

**Authors:** Graeme Fox, Latha R. Vellaniparambil, Loreto Ros, Joshua Sammy, Richard F. Preziosi, Jennifer K. Rowntree

**Affiliations:** ^1^ Department of Natural Sciences, Ecology and Environment Research Centre Manchester Metropolitan University Manchester UK; ^2^ Faculty of Life Sciences The University of Manchester Manchester UK; ^3^ Present address: School of Biological and Marine Sciences University of Plymouth Plymouth UK

**Keywords:** diet, honey bees, landscape, metabarcoding, pollen, urban ecosystems

## Abstract

Growth in the global development of cities, and increasing public interest in beekeeping, has led to increase in the numbers of urban apiaries. Towns and cities can provide an excellent diet for managed bees, with a diverse range of nectar and pollen available throughout a long flowering season, and are often more ecologically diverse than the surrounding rural environments. Accessible urban honeybee hives are a valuable research resource to gain insights into the diet and ecology of wild pollinators in urban settings. We used DNA metabarcoding of the *rbcL* and ITS2 gene regions to characterize the pollen community in *Apis mellifera* honey, inferring the floral diet, from 14 hives across an urban gradient around Greater Manchester, UK. We found that the proportion of urban land around a hive is significantly associated with an increase in the diversity of plants foraged and that invasive and non‐native plants appear to play a critical role in the sustenance of urban bees, alongside native plant species. The proportion of improved grassland, typical of suburban lawns and livestock farms, is significantly associated with decreases in the diversity of plant pollen found in honey samples. These findings are relevant to urban landscape developers motivated to encourage biodiversity and bee persistence, in line with global bio‐food security agendas.

## Graphical abstract

Using 2‐barcode metabarcoding, we identified the floral diet of honeybees across an urban gradient in Greater Manchester. Bees foraging in more urbanized environments had access to a greater diversity of plants than those foraging in more rural environments. Non native and naturalized species comprised an important part of the diet of urban foraging bees along side native species.

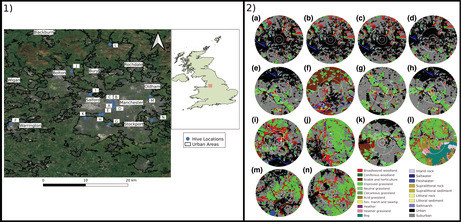

## 1 INTRODUCTION

The highly publicized decline in wild bees, among other pollinators, has been brought into sharp focus for scientists, policy makers, and increasingly for the general public in recent decades (Sánchez‐Bayo & Wyckhuys, 2019; Wagner, 2020). Wild bees and managed honey bees are important components of the pollinator community, providing vital ecosystem services that help sustain our food supply, other crops, and native ecosystems (Hung et al., 2018). As such, a decline in the amount of pollination services provided by wild or managed bees could have serious global consequences (Breeze et al., 2014; Gallai et al., 2009; Klein et al., 2007). Habitat loss and widespread pesticide use are among the most powerful drivers of pollinator decline globally (Gill et al., 2012; Goulson et al., 2015; Jachuła et al., 2022). In the case of honey bees, rates of infection and colony collapse have been attributed to several interacting factors, of which loss of plant forage diversity and abundance has been identified to be an important cause (Branchiccela et al., 2019; Requier et al., 2018; Smith et al., 2013; Vanbergen & The Insect Pollinators Initiative, 2013).

The main sources of nutrition for honey bees are the floral rewards including nectar, which is the primary source of carbohydrate, and pollen, which is the main source of protein, both of which are collected by worker bees (Brodschneider & Crailsheim, 2010). Nutritional requirements of individuals vary by role in the colony, as foragers and nurse bees require different nutrition, and overall foraging intensity is modulated at the colony level (Altaye et al., 2010; Seeley, 1986). Efficient colony maintenance and brood rearing require not only a sufficient quantity of pollen and associated nutrients, but there are also notable benefits associated with a diverse pollen diet. Colonies reliant upon a single monofloral crop, such as those often found in agricultural habitats, experience a brief glut of pollen. However, they may struggle for sufficient nutrition at other times and are particularly susceptible to a failed crop or inclement weather (Dolezal et al., 2019; Topitzhofer et al., 2019; Vanbergen & The Insect Pollinators Initiative, 2013). A diverse diet of plants with flowering times spread throughout the season offers security against these risks, allowing increased temporal stability of nutrient availability (Avni et al., 2014). It also has a direct nutritional benefit compared to a mono‐floral diet, with a diverse diet better able to meet differential nutritional requirements of the different roles within the colony (Paoli et al., 2014). In addition, a poly‐floral diet can increase the immunocompetence of bees and is indirectly associated with an increased number of female offspring and reduced disease and pesticide susceptibility of the colony (Alaux et al., 2010; Centrella et al., 2020). While diet diversity is important for health, it is not the only factor, and total pollen and nectar are also critical. A sufficient biomass of collected pollen is clearly fundamentally important for growth and development, particularly in the spring when stored pollen stocks are low and foraging levels can be inconsistent (DeGrandi‐Hoffman et al., 2016).

Urban intensification and expansion may provide a relatively novel opportunity for wild bees and other pollinators (Ayers & Rehan, 2021; Hall et al., 2017; Turo & Gardiner, 2019; Wenzel et al., 2020). Further increases in the development of urban environments are projected to continue (Chen et al., 2020; Gao & O'Neill, 2020; Seto et al., 2012), and understanding pollinator ecology and behavior in response to changing habitat is necessary. There has been a recent increase in the number of urban beekeepers, with many utilizing gardens and rooftops for their hives (Lorenz & Stark, 2015). Reduced colony mortality, fewer parasitic invasions, and increased colony longevity and reproductive output are all characteristics reported for bee colonies in more urban, compared to rural, environments (Baldock et al., 2019). These benefits are largely attributed to the availability of floral resources and the lower concentration of pesticides (Botías et al., 2017; Samuelson et al., 2018). Agricultural landscapes are often capable of providing comparable or larger quantities of pollen than urban areas, leading to high food accumulation in a hive (Sponsler & Johnson, 2015). However, the diversity of diet is likely to be lower than that collected when foragers are able to access urban landscapes. Urban areas are known to support diverse populations of wild native bees (Baldock et al., 2015; Casanelles‐Abella et al., 2022) alongside honey bees, which like many other bee species, are generalist foragers able to take advantage of the floral diversity available in urban areas. Studying accessible, managed honey bee colonies as models for their wild counterparts is therefore a powerful tool to better understand urban pollinator ecology (Giannini et al., 2015; Lowenstein et al., 2019).

Urban environments, in general, can be considered to be rich in plant diversity, including a mixture of native, those widespread and not introduced by human activity, neophyte, those not native but in the wild and naturalized, and non‐native species, which may include garden plants, recent non‐naturalized escapees, and contemporarily invasive species (Aronson et al., 2017; Baldock et al., 2015; Gaertner et al., 2017; Grimm et al., 2008). This richness is due to the spatial heterogeneity of the areas, which provide niches for opportunistic seedlings, and also the presence of a broad range of cultivated plants in private gardens, parks, allotments, urban food production, and across green infrastructure (Frankie et al., 2005; Garbuzov & Ratnieks, 2014; Knapp et al., 2012; Matteson & Langellotto, 2009).

The degree of urbanization and habitat fragmentation can greatly alter the availability and diversity of floral resources for pollinators (Levé et al., 2019; McKinney, 2002). Even generalist species, such as honey bees, exhibit selectivity in the plant species visited depending upon the needs of the colony at specific times and the availability of resources (Hawkins et al., 2015; Lowenstein et al., 2019; Nottebrock et al., 2017; Requier et al., 2015; Ruedenauer et al., 2020; Salisbury et al., 2015; Vaudo et al., 2015). Foraging distances vary depending on the level of landscape complexity surrounding hives and have been reported to often be shorter in complex and urban or suburban landscapes when foraging for pollen, but the pattern does not continue when foraging for nectar (Garbuzov et al., 2015; Steffan‐Dewenter & Kuhn, 2003). Longer foraging flights of over 9.5 km are known to occur, with foraging strategy theorized to be linked to patch size and quality (Beekman & Ratnieks, 2000).

Plant taxa contributing to the forage of a hive are generally characterized by identification of pollen sourced from hive pollen traps, isolated from honey, or through physically tracking foraging bees (Carvell et al., 2007; Dimou et al., 2006; Valentini et al., 2010). Metabarcoding of DNA; species identification through the analysis of complex, mixed community DNA (Deiner et al., 2017; Hebert et al., 2003; Statnikov et al., 2013), has benefits over methods based on morphology and was popularized for bee forage analysis as DNA barcoding became more prevalent in plants (Dunning & Savolainen, 2010; Kress et al., 2005; Newmaster et al., 2006). While plant species identified from pollen loads give a direct measure of the plants visited by bees when collecting pollen, information from honey‐extracted plant DNA can be used to describe plants visited for both pollen and nectar collection over a longer period (de Vere et al., 2017; Hawkins et al., 2015; Louveaux et al., 1978). Some foraging is known to target pollen only and may therefore be missed when honey‐based sampling is used (Synge, 1947). A number of gene regions (e.g. *rbcL*, *trnL* and ITS2) have been identified for use as metabarcodes in plants and a multi‐gene region metabarcoding approach has been recommended to increase the discriminatory power and broaden the range of species detection, as specific gene regions show biases in detection range and level of plant taxon identification (Bell et al., 2016; Burgess et al., 2011; Hollingsworth et al., 2011; Kress et al., 2009).

In this study, we used DNA metabarcoding of honey samples to determine the diet diversity of honey bees in and around Greater Manchester, UK, and asked how the surrounding landscape composition might influence the diversity and composition of the community of plants visited.

Our aims were to use the data from DNA metabarcoding along with GIS analysis of land cover to address three questions:

1. Does land cover diversity surrounding an apiary predict honey bee diet diversity?
2. Does the proportion of a specific type of land cover surrounding an apiary predict honey bee diet diversity?
3. Does the proportion of urban land cover surrounding an apiary predict the proportions of native, non‐native, and neophyte plants in the honey bee diet?

## 2 MATERIALS AND METHODS

### 2.1 Sample collection

**FIGURE 1:**
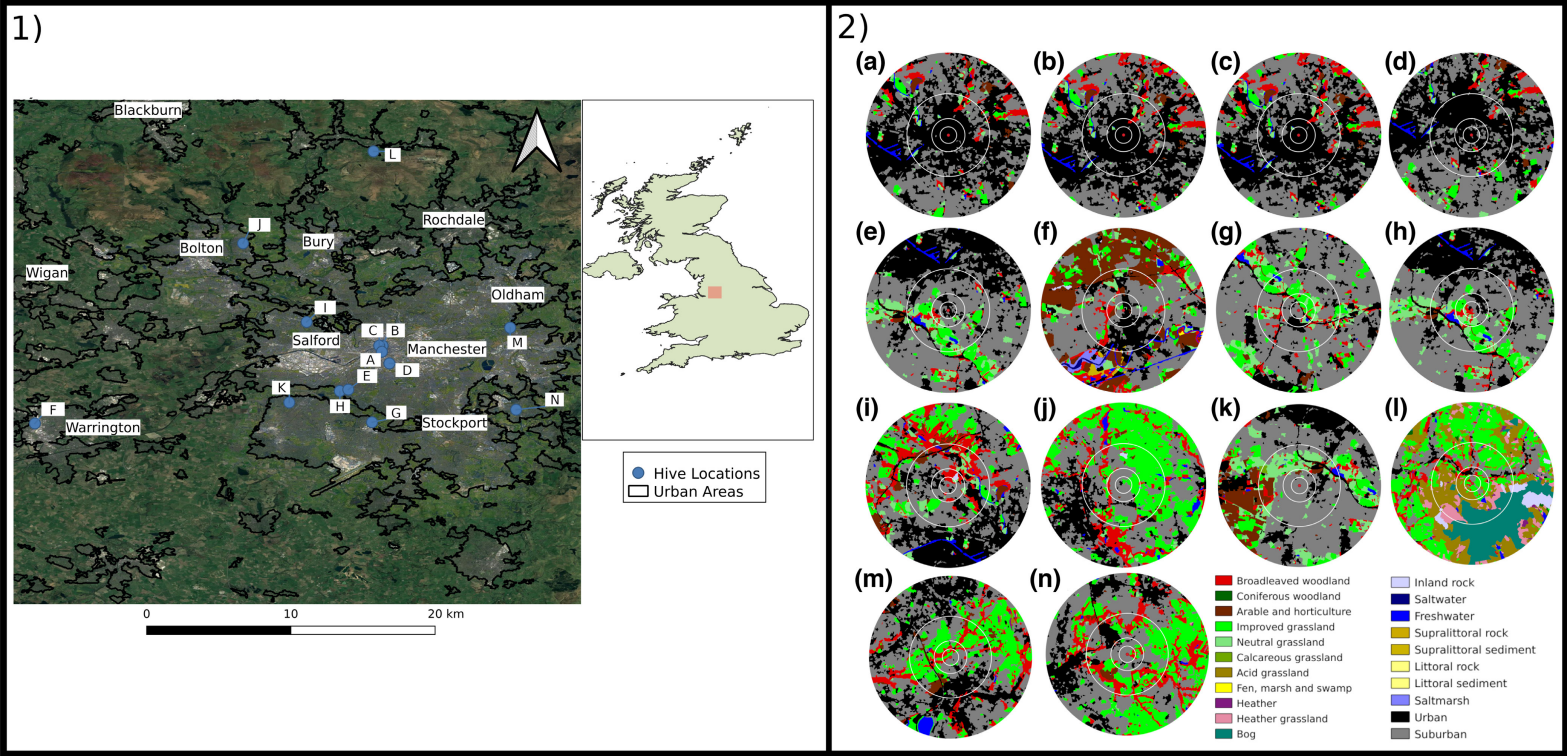
Panel (1) Locations of 14 *Apis mellifera* apiaries (A‐N) from which honey was sampled in the northwest of England. Major towns and cities of the region are labeled. Panel (2) The land cover types surrounding each of the 14 apiaries. Apiaries are located at the central red dot with buffer sizes of 500, 1000, 2500 and 5000 m (outer ring) radius depicted.

Honey samples were sourced from 14 *Apis mellifera* (European honey bee) hives; 12 from Greater Manchester, one from Warrington in Cheshire, and one from Rossendale in Lancashire, across an urban gradient (Figure 1). Extraction of honey varied by the apiary, but the majority of samples were processed with a standard honey extraction method whereby cells are uncapped, the honey is removed from cells by centrifugation in a tangential extractor, and finally filtered to remove large particulates in the honey. Generally, samples were taken from pools of extracted honey collected from multiple frames, but in some instances, comb, chunk, or unfiltered honey were sampled (Table 1). Comb‐honey is honey that has not been removed from the cells, chunk‐honey is a blend of extracted honey and comb‐honey, and unfiltered honey follows the traditional extraction methodology with the omission of the final filtering stage. A single sample was procured from each apiary in 2014/15, although the extraction date is unknown. Honey was sourced from small, independent apiaries that often produce a single harvest per year, with harvest typically occurring in late summer.

**TABLE 1:** Names, abbreviated identifiers, sample type, latitude, longitude, and proportion of urban land cover in the 500‐m, 1000‐m, 2500m‐ and 5000‐m buffers surrounding the postcode of the apiary calculated by GIS analysis of the 14 hives used in the study.

Hive name	Hive code	Sample type	Latitude	Longitude	% Urban land cover (500 m)	% Urban land cover (1000 m)	% Urban land cover (2500 m)	% Urban land cover (5000 m)
Art Gallery	A	Trad.	53.478848	−2.241449	100.0	99.0	69.0	49.0
Printworks	B	Trad.	53.485179	−2.240747	99.2	96.3	65.0	47.2
Manchester Cathedral	C	Trad.	53.485109	−2.244227	98.5	93.1	65.0	47.3
Manchester Museum	D	Trad.	53.466753	−2.233674	84.7	75.3	67.0	48.0
Chorlton	E	Chunk	53.439583	−2.276330	55.0	28.0	21.3	32.0
Warrington	F	Trad.	53.404419	−2.601781	48.1	38.5	30.2	13.6
Northenden	G	Trad.	53.405705	−2.251699	18.8	2155	12.3	14.5
Chorlton Meadow	H	Trad.	53.438089	−2.285144	16.3	21.6	21.1	30.2
Pendlebury	I	Trad.	53.509781	−2.319582	14.6	18.0	21.0	27.2
Bury	J	Trad.	53.591346	−2.385633	5.9	2.2	8.9	15.0
Sale	K	Comb	53.426212	−2.337615	1.8	8.7	9.1	19.0
Cowpe	L	Trad.	53.686795	−2.250133	0.8	6.7	3.4	2.5
Oldham	M	Unfiltered	53.503820	−2.108313	0.6	6.3	20.0	27.3
Stockport	N	Trad.	53.418710	−2.102141	0.2	9.4	10.6	16.0

*Note*: Sample type refers to the process by which the honey was collected from the hive with traditional methods referring to the standard multi‐frame method used by beekeepers.

### 2.2 Landscape analysis

The hive locations were identified by postcode of apiaries, at the request of the beekeepers. Latitude and longitude were derived based upon the full UK postcode and therefore have a limited precision (an average UK postcode covers an area of approximately 0.135 km^2^). Locations of apiaries were mapped using QGIS v.2.14.0 (*QGIS Geographic Information System*, 2020) based on the geographic coordinates (Table 1). To adequately describe land cover across a range of spatial scales most typically used by foraging bees (Garbuzov et al., 2015; Sponsler & Johnson, 2015), the landscape surrounding each apiary was characterized using buffers with radii of 500, 1000, 2500, and 5000 m. The majority of foraging flights for both pollen and nectar occur within a radius of ≤5000 m, although less foraging behavior has been known to occur further afield when necessary (Beekman & Ratnieks, 2000; Couvillon et al., 2015). The GB 25m raster land cover data set for the study area was obtained that identifies 21 landscape classes (Rowland et al., 2017). The proportion of each of the 21 land cover class at the different spatial scales was determined using LECOS (Jung, 2013), a QGIS plugin for calculating patch‐based landscape metrics (Tables 1 and 2 and Figure 1). To test for associations between proportions of land cover types in the buffer zones, a Spearman's correlation coefficient was calculated for each pair using the R “psych” package (Revelle, 2017).

**TABLE 2:** Details of the LCM2015 land cover map land classes used in the analysis

LCM2015 class number	Land use abbreviation	Land use name
1	Broadleaved woodland	BW
2	Coniferous woodland	CW
3	Arable and horticulture	AH
4	Improved grassland	IG
5	Neutral grassland	NG
6	Calcareous grassland	CG
7	Acid grassland	AG
8	Fen, marsh, and swamp	FS
9	Heather	HR
10	Heather grassland	HG
11	Bog	BO
12	Inland rock	IR
13	Saltwater	SW
14	Freshwater	FW
15	Supra‐littoral rock	SLR
16	Supra‐littoral sediment	SLS
17	Littoral rock	RO
18	Littoral sediment	LS
19	Saltmarsh	SM
20	Urban	UB
21	Suburban	SB

### 2.3 Total DNA extraction

Total DNA was extracted from 40 g honey using a modified protocol for the DNeasy Plant Mini Extraction Kit (Qiagen) described in Hawkins et al. (2015). Each honey sample was homogenized by stirring with a sterile stirrer. Four subsamples of 10 g were diluted in 25 ml molecular biology–grade H_2_O (Sigma Aldrich) and incubated at 65°C with periodic shaking. Once completely dissolved, each tube of dissolved honey was centrifuged at 15,000 *g* for 30 min and the supernatant discarded. Parallel centrifugation of the subsamples allows for a larger volume and therefore quantity of honey and pollen to be effectively sampled. Dilution in H_2_O reduced the specific gravity of the resulting solution sufficiently for pollen to be concentrated in a high‐speed centrifuge. The ratio of honey:water is comparable to that in other recent honey metabarcoding research (Jones et al., 2021). The pellet was suspended in 400 μl AP1 buffer (Qiagen DNeasy Plant Mini Extraction Kit) and 4 μl proteinase K added (20 mg/ml) (Bioline). To mechanically disrupt the pollen, two 3‐mm tungsten carbide beads (Qiagen) were added to each sample prior to processing (4× 1 min cycles at 30 Hz) with a Retsch MM400 mixer mill (Retsch) before a further incubation at 65°C for 30 min. Each set of four subsamples were pooled in a single DNeasy Plant Mini kit spin column (Qiagen), and the extraction continued as directed by the manufacturer's manual but with the omission of the QIA shredder column step and the second wash stage (AW2 wash buffer). Extracted DNA was frozen for long‐term storage at −80°C.

### 2.4 DNA amplification and sequencing

DNA was amplified using two sets of PCR primers, one amplifying the chloroplastic *rbcL* gene (Hollingsworth et al., 2009) and one amplifying a plant‐specific variant of the internal transcribed spacer region 2 (ITS2) of the nuclear ribosomal region (Chen et al., 2010). Herein, they are referred to as *rbcL* and ITS2 pLant (ITS2p), respectively (Table 3). Control amplifications using negative control DNA extractions as the DNA template produced no visible bands on 2% electrophoresis gels and were not progressed further. The PCR products were prepared for sequencing using a two‐stage PCR protocol detailed in the Illumina *16S* Library Preparation workflow adapted for use with non‐*16S* regions. The initial PCR amplifies the region of interest using locus‐specific primers to which the 5′ Illumina adapter overhangs have been added. This PCR used a final volume of 25 μl:2 μl template DNA; 12.5 μl of 2× KAPA HotStart Ready Mix; 0.5 μl (10 μM) forward primer; 0.5 μl (10 μM) reverse primer, and 9.5 μl of molecular biology–grade H_2_O. Samples were amplified by an initial denaturation at 95°C for 3 min, 30 (*rbcL*) or 27 (ITS2p) cycles of denaturation (98°C for 20 s), annealing (*rbcL*: 60°C, ITS2p: 62°C for 30 s), and extension (72°C for 30 s), with a final extension (72°C for 10 min). The PCR products generated were submitted to the Centre for Genomic Research (CGR) at the University of Liverpool where the remaining steps in the workflow were completed (inclusion of Illumina Nextera molecular identifiers by a second round of PCR and pooling in equimolar concentrations) and sequenced on a lane of an Illumina HiSeq 2500 as a 2 × 300 bp rapid run using a V2 flowcell. Raw DNA sequence data are available from the N.C.B.I. sequence read archive under accessions SRR16143791 to SRR16143818.

**TABLE 3:** Details of PCR primers used to amplify the two metabarcoding gene regionsmarkers.

Gene regionmarker	Primer Name	Sequence (5′–3′)	Reference
*rbcL*	F: *rbcLa‐F* R: *rbcLr590*	ATGTCACCACAAACAGAGACTAAAGC AGTCCACCGCGTAGACATTCAT	Hollingsworth et al. (2009)
ITS2p	F: S2F R: S3R	ATGCGATACTTGGTGTGAAT GACGCTTCTCCAGACTACAAT	Chen et al. (2010)

### 2.5 DNA sequence data analysis

Low‐quality regions of sequence data, along with very short reads, were removed with Trimmomatic (parameters used: LEADING:3 TRAILING:3 SLIDINGWINDOW:4:30 MINLEN:50) (Bolger et al., 2014) and adapters removed using Cutadapt (Martin, 2011). Denoising of sequence data, removal of chimeric sequences, and generation of amplicon sequence variants (ASVs) were performed using the DADA2 (Callahan et al., 2016) functionality in the QIIME2 package (Bolyen et al., 2019). Taxonomic assignment of ASVs was performed by the naïve Bayesian classifier in QIIME2 using the “q2‐feature‐classifier” Python script, against either the “*rbcL* reference library” (https://doi.org/10.6084/m9.figshare.c.3466311.v1) or “Pollen/Plant ITS2 reference set for the RDP/UTAX classifier (2015)” database (Bell & Brosi, 2016; Sickel et al., 2015) as appropriate, against which the classifier had previously been trained. Any ASVs assigned a taxonomy outside the plant kingdom were removed. Any ASV lacking species‐level resolution was searched against the N.C.B.I. nucleotide database using megablasts for highly similar sequences (Camacho et al., 2009). Species‐level taxonomy was assigned where the best match was achieved against a voucher specimen of a single species. If multiple species in the same genus were equally probable, then genus‐level taxonomy was assigned. If multiple genera were equally probable, then family‐level taxonomy was assigned, and so on. In the data of each gene region, ASVs with identical taxonomic assignments were collapsed together and ASV counts combined.

Low‐frequency incidences of collapsed ASVs were removed from individual samples; where the percentage of reads associated with an ASV in a single sample was <0.03% of total reads associated with that ASV. Low‐frequency ASVs within each sample (<1% of total sample reads) were also subsequently removed (Taberlet et al., 2018).

### 2.6 Pollen taxonomy

Species or genus assignments were checked for plausibility of their presence in the UK against several databases: the Royal Horticultural Society Horticultural Database (Royal Horticultural Society, 2018) based upon the BG‐BASE database v7.3 (BG‐BASE Collections Management Software, 2018); the Biological Records Centre Atlas of the British and Irish Flora (Biological Records Centre, 2018); and the Plants For A Future database (Plants For A Future, 2018), and against a guide to British flora (Stace, 2010) (Appendices A, B, C). Species missing from these sources were checked for availability in online, UK‐based garden centers and assigned UK plausibility accordingly. Genus‐level assignments were filtered based on the plausibility of the genus being present in the UK based on the same records as the species‐level data. Implausible taxa were removed, and abundance matrices from the two gene regions were combined into a single, unweighted presence–absence matrix to maximize the detection range. Only ASVs achieving genus‐level assignment or better were retained. Species‐level assignments were assigned a category based upon their status in the UK, as either native, non‐native, or neophyte.

### 2.7 Statistical analysis

All analyses were performed using R (version 4.0.4) (R Core Team, 2021), in the RStudio environment (version 2021.09.0, Build 351) (RStudio Team, 2021), all plots produced using “ggplot2” (Wickham, 2016) and FDR corrections performed using the “p.adj” function in the base R package “stats.” QIIME2 output was imported into R using the package “qiime2R” (Bisanz, 2018). The R package “vegan” (Oksanen et al., 2020) was used to calculate rarefaction curves and Shannon diversity indices of plant taxa and land cover in buffers. Fisher's exact test was used to test the independence of the apiary from proportions of native, neophyte, and non‐native species. To test for differences between native, neophyte, and non‐native groups, one‐way ANOVAs upon the proportions of each, per hive were run and subsequently the pairwise group means tested for significant differences using the Tukey HSD posthoc test from the R package “stats” (R Core Team, 2021). For investigation into the relationships between types and diversity of plants in the honey and land cover surrounding an apiary, we elected to focus on the 5000‐m buffer surrounding each apiary, which covers the majority of foraging flights. Proportions of native, non‐native, and neophyte plants were tested for significant associations with proportion of urban land cover in the 5000‐m buffer through calculation of Pearson's correlation coefficient. Pearson's correlation coefficient was used to describe the relationship between Shannon diversity of the plant taxa in each honey and the Shannon diversity of land cover surrounding each hive in the 5000 m buffer as well as the relationships between Shannon diversity of plant taxa in each sample and the proportions of each land cover type in the 5000‐m buffers.

## 3 RESULTS

### 3.1 Land cover composition around hives

Of the 21 landscape components defined in the UK Landcover map 2015, 11 classes were identified in the 500‐m and 1000‐m buffer zones around at least one of the 14 hives studied. This increased to 16 classes in the 2500‐m and 18 classes in the 5000‐m buffer zones. The most common landscape components captured were urban, suburban, neutral grassland, improved grassland, and broadleaved woodland. Four hives were located in Manchester city center with >84% urban land cover in the 500‐m buffer and >47% urban in the 5000‐m buffer (Table 1). One hive (A) was represented by a single class (urban) within the 500‐m buffer zone. The remaining hives were distributed across Greater Manchester and surrounding area with increasing proportions of suburban, natural and semi‐natural grasslands, and broadleaf woodland as distance from the city center increased (Figure 1). The predominant classes within the 500‐m buffer zones were urban and suburban, except for one hive (L) which had improved grassland as the dominant class. Proportions of land cover types were mostly independent of one another. In the 500‐m buffers, we observed a significant negative correlation between proportions of SB (suburban) and UB (urban) (*r*_[12]_ = −.83, *p* = .01). In the 5000‐m buffers, there were no significant associations between classes of land cover.

### 3.2 Plant community composition

Per‐apiary, mean *rbcL* reads passing quality trimming and denoising were 120,773.6 (SD: 59,371.8) and mean ITS2p reads were 345,796.4 (SD: 130,395.2). The UK plausibility filter removed 1.17% reads of these reads assigned to a species and 0.56% reads assigned to a genus only in *rbcL* and 0.43% assigned to a species and 0.64% assigned to a genus only in ITS2p. After removal of very low‐frequency taxa, 24 species and 42 genera plausible to be present in the UK were detected across all honey samples (Appendixes D and E). In *rbcL*, 95.08% of reads were assigned a genus and 56.60% assigned a species, while in ITS2p, 84.51% were assigned a genus and 46.50% were assigned a species. The number of unique taxa per hive ranged from 3 to 21. Species‐level identification was possible for 40% of ASVs. Rarefaction curves showed that every sample had reached a detection plateau, sufficient for confident detection of all taxa. Diversity varied among hives (Appendix F), with the two least diverse samples characterized by the presence of a single species (*Impatiens glandulifera*) and two unresolved genera. Shannon diversity ranged from a minimum of 1.1 to a maximum of 3.14 (Table 4). Taxonomic resolution differed between the two gene regions with entire genera unable to be resolved to species in both. Due to the different specificities of each gene region, only *Hydrangea* and *Juglans* were unable to be classified to species level in either region, and as such the combination of data from both regions gives broad spectrum taxonomic detection. Unspecified *Hydrangea* spp. and *Juglans* spp. reads accounted for 0.97% and 0.37% of raw reads, respectively.

**TABLE 4:** Shannon diversity indices for each of the 14 hives using taxa derived from both rbcL and ITS2p.

Hive	Shannon diversity
A	3.04
B	2.56
C	2.40
D	3.00
E	2.20
F	2.08
G	2.64
H	3.14
I	2.20
J	1.79
K	2.30
L	1.10
M	1.10
N	1.79

Considering the ASVs with species‐level assignment, native, non‐native, and neophyte species were each found in most, but not all hives, and the number of species in each category was independent of the hive (Fisher's exact test, *p* = .9984). There were significant differences between each group as determined by one‐way ANOVA (*F*_2,39_ = 11.4, *p* = .0001) with Tukey HSD pairwise post‐hoc tests revealing a significant difference between native and neophyte species (*p* = .0001, 95% C.I. = 0.19, 0.59), with neophytes exceeding natives, and a significant difference between non‐native and neophyte species (*p* = .022, 95% C.I. = 0.03, 0.43), with neophytes exceeding non‐native but no significant difference between native and non‐native species (*p* = .133, 95% C.I. = −0.04, 0.36).

The most common plant species was *Impatiens glandulifera*, a neophyte, which was found in honey from every apiary. Also very common were *Olea europaea* and *Rubus armeniacus*, both non‐natives, and *Trifolium repens*, a native, as well as *Impatiens* spp. and *Rubus* spp. which did not achieve species‐level resolution. These common ASVs were all found in honey from >50% apiaries. Other trees and shrubs detected included those frequently found in towns and cities such as *Quercus* spp., *Tilia* spp., *Malus* spp., *Buddleja* spp., *Prunus* spp., *Caragana* spp., *Salix* spp., *Hydrangea* spp., and *Sorbus* spp. Another taxon of note is *Cannabis sativa* which was detected in a single honey. Raw sequence counts assigned at genera and species in each gene region, along with appropriate metadata, are found in the appendices.

**FIGURE 2:**
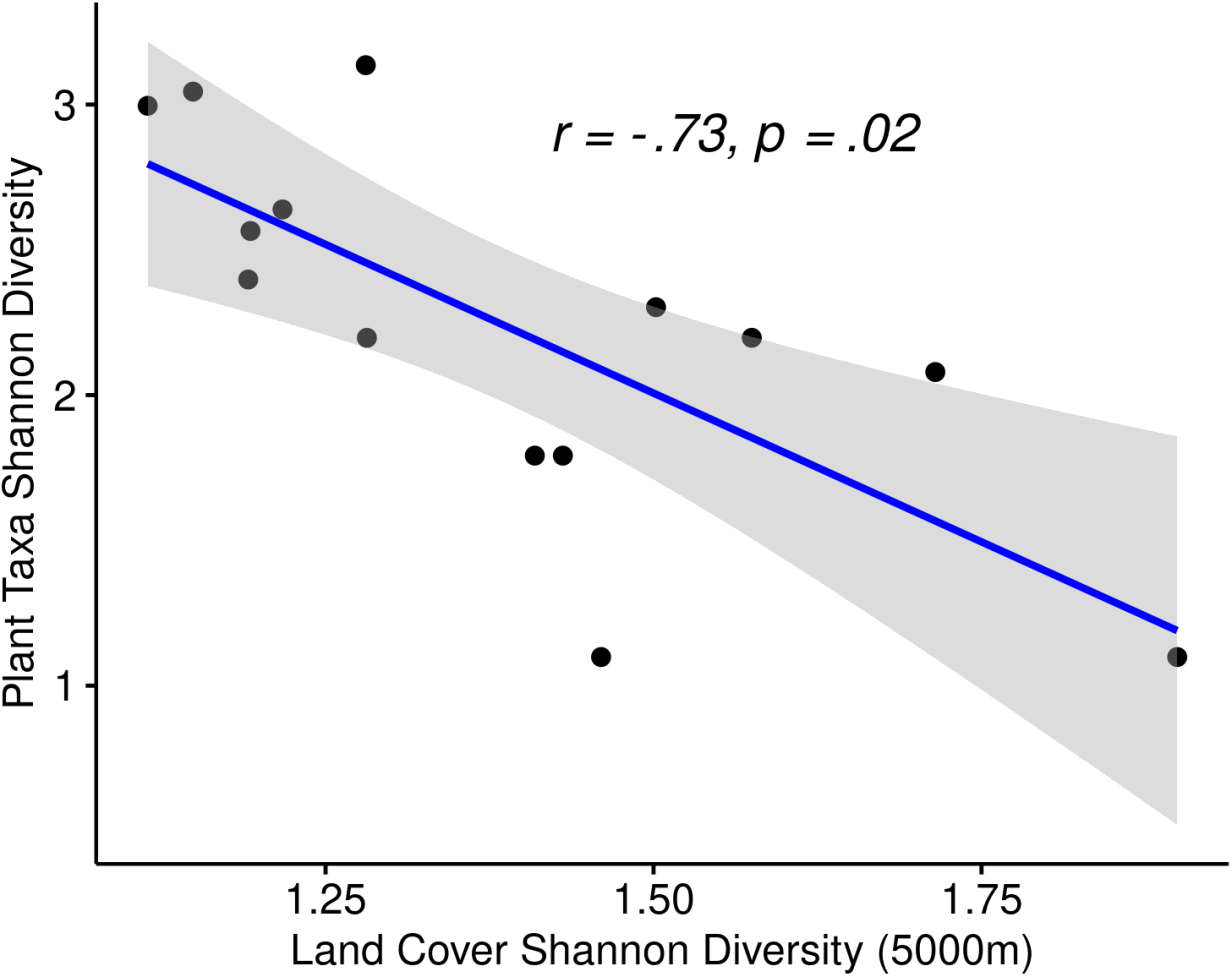
Shannon diversity of plant taxa within a hive is significantly negatively correlated with Shannon diversity of land cover in the surrounding 5000 m, a distance likely to cover the majority of foraging flights by bees. Each data point represents a single hive. Pearson's correlation coefficient is presented along with the linear regression line. The shaded area represents the 95% confidence interval.

A significant negative relationship was observed between Shannon diversity of plant communities in honey and Shannon diversity of the surrounding landscape in the 5000‐m buffers (*r*_[12]_ = −.73, *p* = .02) (Figure 2). Plant communities include all taxa detected, including those ASVs achieving only genus‐level assignment.

**FIGURE 3:**
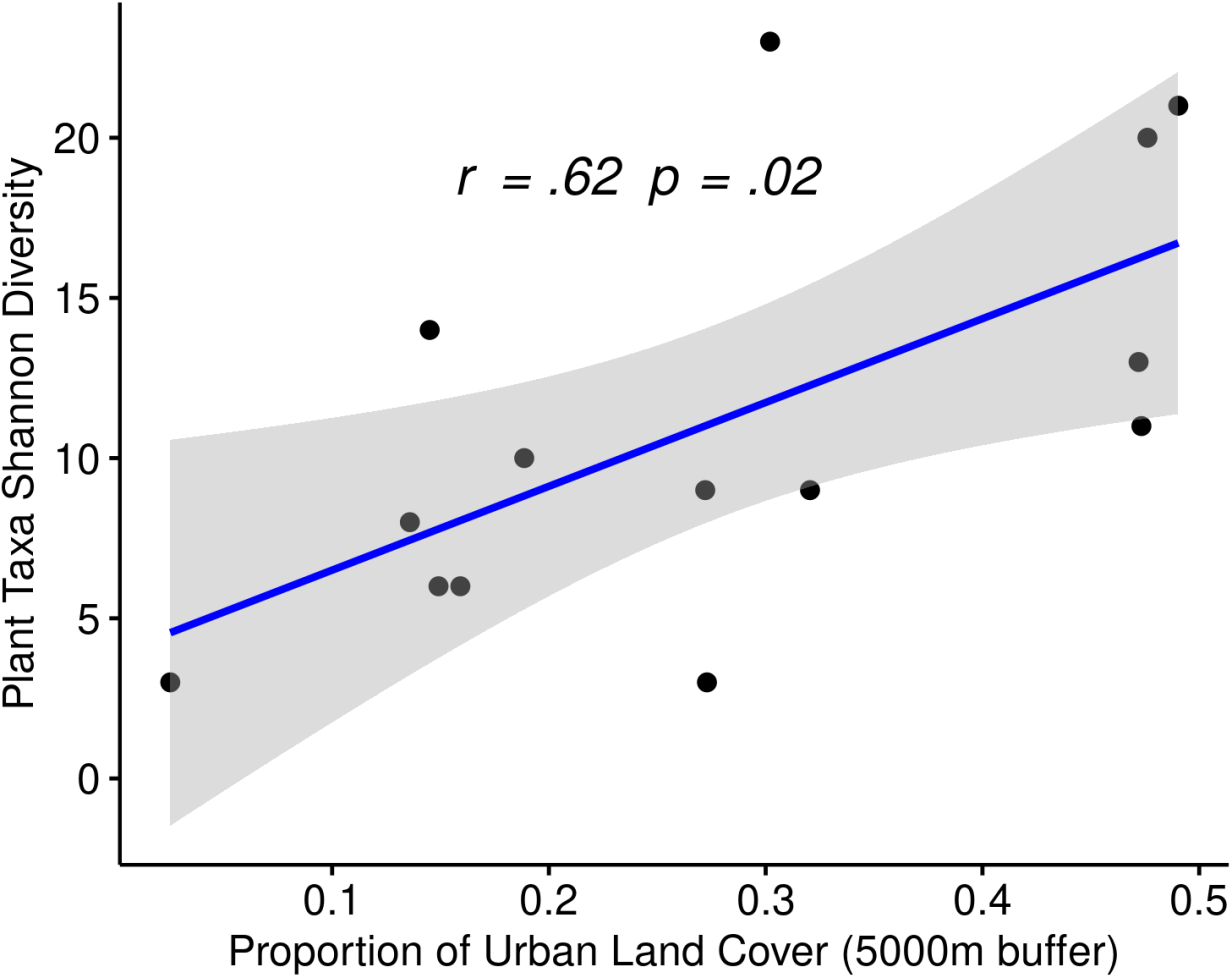
Proportion of urban land in the 5000‐m buffer surrounding a hive (a distance likely to cover the majority of foraging flights by bees) is significantly positively correlated with the Shannon diversity of the plant taxa detected in the honey of the hive. Pearson's correlation coefficient is presented along with the linear regression line. Each data point represents a single hive. The *p*‐value has been adjusted to control for false discovery. The shaded area represents the 95% confidence interval.

**FIGURE 4:**
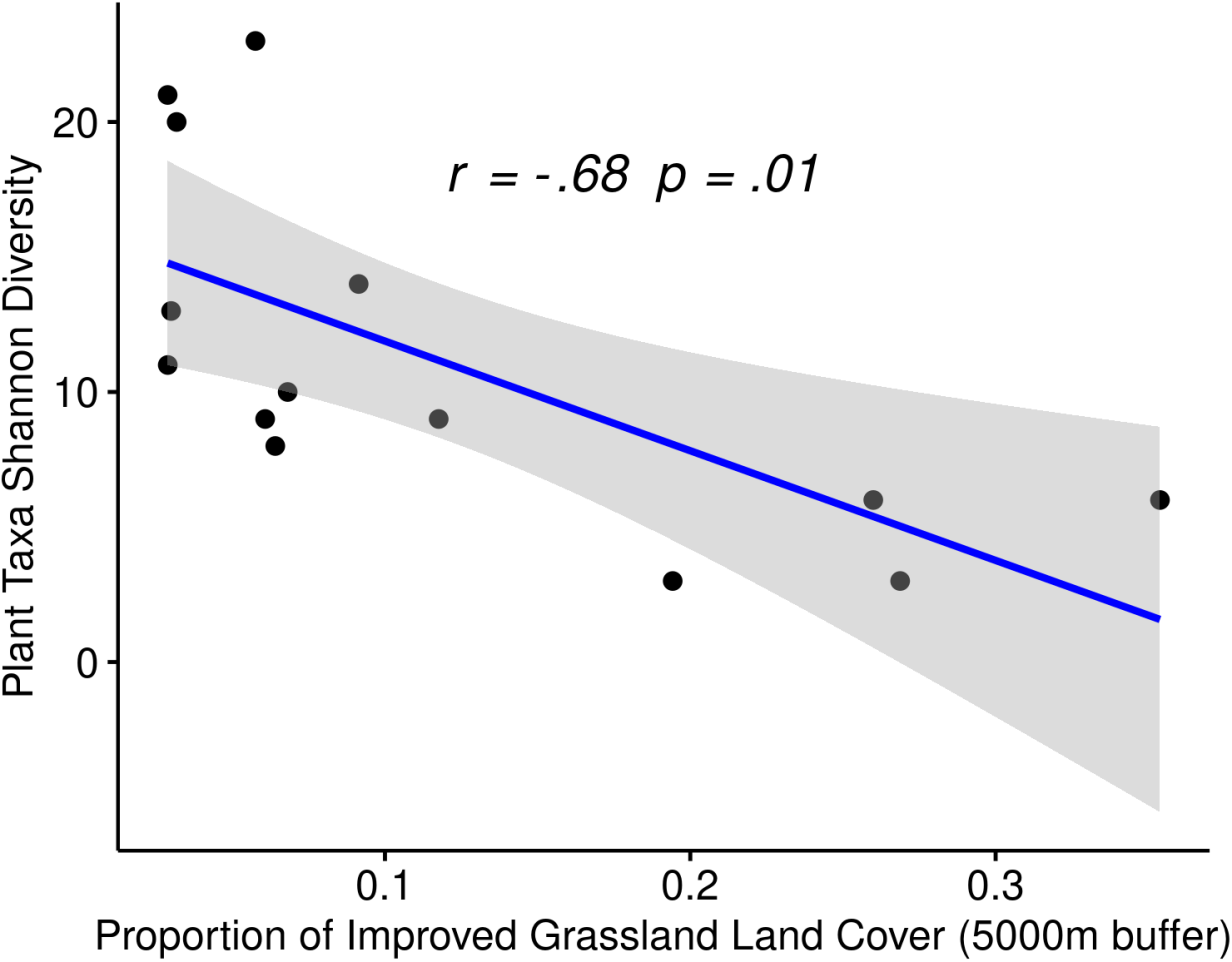
Analysis of the relationship between the proportion of improved grassland (IG) in the 5000‐m buffer surrounding a hive (a distance likely to cover the majority of foraging flights by bees) and the Shannon diversity of the plant taxa detected in the honey showed a significant negative correlation. Pearson's correlation coefficient is presented along with the linear regression line. The *p*‐value has been adjusted to control for false discovery rate. Each data point represents a single hive.

A significant positive association was observed between plant diversity and the proportion of urban land cover in the 5000‐m buffers surrounding hives (*r*_[12]_ = .62, *p* = .02) (Figure 3). Furthermore, a significant negative association was reported between the proportion of improved grassland and plant diversity (*r*_[12]_ = −.68, *p* = .01) (Figure 4). Urban land cover typically includes town and city centers of very little vegetation cover and also includes areas such as docks, car parks, and industrial estates. Improved grassland includes high production grassland, characterized by a lack of winter senescence and is sometimes heavily grazed.

The proportion of urban land cover in the 5000‐m buffers was not significantly associated with the proportions of native, non‐native, and neophyte plant species detected in the diet. The proportion of urban land cover in the 500‐m buffer was significantly positively associated with the proportion of native species (*r*_[12]_ = .71, *p* = .02) and marginally significantly negatively correlated with neophyte species (*r*_[12]_ = −.61, *p* = .08). There was no significant relationship between the proportion of urban land cover and the proportion of non‐native plants (*r*_[12]_ = .37, *p* = .48).

## 4 DISCUSSION

We used next‐generation DNA sequencing and a bioinformatics workflow to describe plant taxa in honey and analyzed the plant community data in the context of land cover in the areas surrounding 14 *A. mellifera* hives. Diverse wild pollinator populations are common in heavily urbanized areas (Baldock et al., 2015; Casanelles‐Abella et al., 2022) and our findings suggest that wild bees are likely to have access to a diverse diet in these areas, alongside managed honey bees. Further work to investigate the influence of land cover on wild generalist bees would undoubtedly be beneficial to support their conservation and to inform efforts to manage some of the global threats to pollinators. While our findings suggest urban areas are a valuable resource for wild bees and honey bees alike, among other pollinators, we are not promoting further uptake in urban beekeeping. Managed *A. mellifera* colonies can transfer parasites to wild bee colonies and are known to compete with wild bees for resources, potentially mitigating the opportunity that urban areas present and even further exacerbating their declines (Goulson & Sparrow, 2009; Pirk et al., 2017).

### 4.1 Influence of landscape components on honey bee diet

We found plant taxa richness to be significantly negatively correlated with diversity of land cover surrounding apiaries. Habitat heterogeneity theory tells us that a larger, more heterogeneous environment provides a greater number and wider variety of available habitats or niches and is therefore likely able to support a more diverse flora and fauna (Kallimanis et al., 2008) in apparent contradiction to this finding. In terms of diversity of plant forage available, highly heterogeneous urban landscapes can, in some instances, host more diverse plant communities than landscapes consisting of more diverse but homogenous, land cover types. In this, they can offer an attractive refuge for a diverse community of bees and other pollinators (Daniels et al., 2020; Hall et al., 2017; Hülsmann et al., 2015; Kowarik, 2011; Lowenstein et al., 2019; Somme et al., 2016; Theodorou et al., 2020). This is further supported by our findings as we recorded a significant positive association between the proportion of urban land cover surrounding apiaries and the diversity of plants detected in the honey. Manchester city center has a disproportionately large number of high‐density residential properties (62.6% of all housing in the city) (Baker et al., 2018), and we see that in other comparable cities, the presence of many smaller gardens, cultivated or left wild, provide a diverse forage for bees (Gaston et al., 2005; Lowenstein & Minor, 2016). The post‐industrial cityscape also contains many brownfield sites described as being characteristically long‐term derelict, vacant, and/or contaminated (Dixon et al., 2010), as well as verges, canal towpaths, and other unmanaged areas. Unmanaged areas, urban meadows, and private gardens are very often occupied by native “weed” species, many of which are highly prized sources of pollen and nectar (Sponsler & Johnson, 2015; Turo & Gardiner, 2019; Weaver, 1965). These species often provide their floral rewards either very early or very late in the bee foraging season, providing high value nutrition when forage availability might otherwise be low (Hicks et al., 2016). Garden escapees, alongside wild opportunistic seedlings in urban areas, have the added advantage of being unlikely to be treated with pesticides common in agricultural, horticultural, and floricultural trades (Goulson et al., 2018; Lentola et al., 2017). Studies in other urban areas have shown higher plant diversity in the private gardens of diverse areas with both ornamental and weed species contributing to the complexity, and there is ample scope for high‐density vertical planting (green walls, planters on balconies of high‐rise buildings) alongside relatively little use of ornamental lawns in highly urbanized municipal planting schemes and domestic gardens (Aronson et al., 2017; Knapp et al., 2012; Lowenstein & Minor, 2016). Several studies now show that urban and suburban environments appear to support a greater diversity of pollen in the diet than that provided by other surrounding land cover types (Lucek et al., 2019; Richardson et al., 2021). Our study adds to and supports this body of work, expanding it to include other sites.

Every hive in our study had multiple types of land cover in the surrounding buffers, with larger buffers more likely to capture a diverse range of land cover types. As such, even those hives in which the smallest (500 m) buffer was dominated by a single land cover type, the proportion of that dominant type was reduced at larger buffers. In the cases of four hives with very high proportions of urban land cover in the 500‐m buffer, the proportion of urban land decreased as the buffer size around the hive increased. Foragers from these hives, therefore, have ample scope to access other types of land cover on foraging flights, most commonly suburban areas, the secondary land cover type surrounding these hives. Previous studies have demonstrated that urban bees in the UK can access ample floral resources at close proximity and forage mostly within a smaller range (500 m to 1.2 km) than the maximum distances recorded by foraging bees (~12 km); however, this does appear to be seasonal and not universal to all urban areas (Beekman & Ratnieks, 2000; Garbuzov et al., 2015; Sponsler & Johnson, 2015; Steffan‐Dewenter & Kuhn, 2003).

Honey sampling is recognized to provide detection of plants over a broad temporal range and here enabled the detection of a wide range of plants (de Vere et al., 2017; Hawkins et al., 2015; Louveaux et al., 1978). It is reasonable to argue that our analysis describes forage collected in highly urbanized areas at some point of the foraging year, but we are unable to describe the foraging range of bees from these hives conclusively. The pooling of honey from multiple combs during a traditional extraction, albeit from within an apiary, will very likely increase pollen diversity in a given sample due to honey being made at different stages of the foraging season. While our samples were a mixture of extraction types, removal of non‐traditional (single frame) samples did not yield any notable differences in our results, and as such all samples were included in analyses. The patterns we report in the data may well be specific to samples collected at a particular time of the foraging season, as forage diversity is known to vary throughout the year (Requier et al., 2015). Further sampling of honey from similar hives across the temporal range of the foraging period would further elucidate the apparent relationship between diet diversity and surrounding land use.

We have shown a significant negative relationship between the proportion of improved grassland surrounding an apiary and the diversity of plants in the honey. The hive with the highest proportion of improved grassland in the 500‐m buffer (L) is by far the most rural in the simple terms of proximity to a large town or city and has one of the smallest proportions of urban land in its immediate surroundings (0.8% UB in 500 m buffer). This particular hive showed very low plant diversity, containing only *I. glandulifera*, *Impatiens* spp. (very likely *Impatiens glandulifera*, but species resolution not possible for this ASV), and *Rhododendron* spp., all of which are known to be rich sources of pollen (Hicks et al., 2016). Clearly absent from this rural hive are some of the woody species that make up a large component of the diet of other hives. Notable by their absence are the Oleaceae, Fabaceae, Fagaceae, and Brassicaceae, which although not uniformly present in every other hive, are all common families across the data set. The reliance of bees from this rural hive on a very small number of taxa is of concern. The loss of flower resources due to farming intensification is recognized as an important driver in pollinator declines (Potts et al., 2010). Furthermore, the importance of the introduction or restoration of flower‐rich habitats in improved grasslands in order to enhance biodiversity for pollinators has also been established previously (Orford et al., 2016).

### 4.2 Plant metabarcoding technical considerations

We found that the combination of laboratory and bioinformatics methods employed produced many false positive results at the taxon assignment stage, in common with many metabarcoding studies (Ficetola et al., 2016; Porter & Hajibabaei, 2018; Zinger et al., 2019). For example, the data described plant species in our samples that were unlikely to be growing in the region. A database of plausible taxa in the ecosystem is therefore invaluable for quality control and should be generated and evaluated with the highest possible level of stringency. In the British Isles, we have an extremely well‐characterized and barcoded native flora (Ratnasingham & Herbert, 2007; Stace, 2010), but the range of plants visited by bees and other pollinators is often much more diverse. A generalist forager such as a honey bee will forage exotic cultivars in gardens, and some invasive species are known to provide the majority of nectar and pollen to a hive when available (Donkersley et al., 2017), as we found in the present study. Where research is focused upon urban and suburban ecosystems, in particular, it is important to adjust the criteria by which plausible plant taxa are filtered, as a simple filter which only passes native, or naturalized, plant species will not suffice. Initial exploratory analysis of the data revealed that implausible taxonomies were much more likely to be assigned to ASVs present at extremely low relative abundances. This is potentially due to errors inserted at a low frequency into amplicons during PCR amplification, DNA sequencing or as other artifacts of the data analysis method. To handle these low‐frequency, implausible taxa, we removed any low‐frequency ASVs during data processing (Taberlet et al., 2018). After this blunt‐edged, but highly effective, data processing step, relatively few implausible ASVs remained, and any that remained were also subsequently removed by our filter against the database of plausible taxa.

## 5 CONCLUSION

The combination of DNA metabarcoding and GIS analyses provides a powerful tool to describe the influence of land cover on the pollen diet of bees. The managed *A. mellifera* colonies are a valuable resource for wider pollinator research and can also be used as a model to infer important concepts to the conservation of wild bees. Urban environments can provide an abundant and diverse pollen diet, suitable for a generalist pollinator such as *A. mellifera*, as well as other wild pollinators. The highly heterogeneous habitats characteristic of urban settings provide ample opportunities for a diverse array of pollen‐ and nectar‐rich plants including native, non‐native, and neophyte species. Improved grassland, including the lawns typical of suburban habitats and of agricultural environments, provides forage with far less plant diversity. These pollen‐poor areas could be improved by allowing the common lawn weeds *T. repens*, *T. officinali*, or *B. perennis* to prosper and flower. In farm agri‐environmental schemes, this often takes the form of strip planting of pollen‐ and nectar‐rich flower mixes in edges and the improvement of grassland by the introduction of legumes such as the *Trifolium* spp. (Carreck & Williams, 2002; Requier et al., 2015; Wood et al., 2015). Given the previously stated ecological and financial importance of bees and other pollinators, the relative merits of urban and agricultural spaces as providers of a healthy diverse forage are not in balance. Considering our samples and analyses, we conclude that urban spaces currently represent a valuable, diverse pollen and nectar resource for pollinators. While a valuable member of the global pollinator community, *A. mellifera* are not under threat and although urban colonies such as those in the present study can be a valuable research resource, increasing density of apiaries may in fact negatively impact wild bees further (Goulson & Sparrow, 2009; Pirk et al., 2017). Non‐native and neophyte species play a critical role in expanding the diet diversity beyond native species and also in increasing the foraging season, allowing the generation of large honey reserves for the colony.

## AUTHOR CONTRIBUTIONS

**Graeme Fox:** Conceptualization (supporting); formal analysis (lead); investigation (equal); methodology (equal); project administration (supporting); writing – original draft (equal); writing – review and editing (lead). **Latha R. Vellaniparambil:** Conceptualization (supporting); formal analysis (supporting); funding acquisition (equal); investigation (equal); methodology (equal); project administration (supporting); writing – original draft (equal); writing – review and editing (supporting). **Joshua Sammy:** Investigation (supporting); methodology (supporting); writing – review and editing (supporting). **Loreto Ros:** Investigation (supporting); methodology (supporting); writing – review and editing (supporting). **Richard F. Preziosi:** Conceptualization (supporting); formal analysis (supporting); funding acquisition (equal); supervision (supporting); writing – review and editing (supporting). **Jennifer K. Rowntree:** Conceptualization (lead); formal analysis (supporting); funding acquisition (equal); project administration (lead); supervision (lead); writing – original draft (supporting); writing – review and editing (supporting).

## CONFLICT OF INTEREST

The authors declare no conflicts of interest.

## Data Availability

Next‐generation sequencing data are available for public download from the N.C.B.I sequence read archive (SRA) database under identifiers SRR16143791 to SRR16143818. The associated R analysis script, intermediary data files, plant metadata, and QGIS land use data are all available to download from GitHub (https://github.com/graemefox/Mancester_honey) and from Dryad (DOI: https://doi.org/10.5061/dryad.5hqbzkh91).

## APPENDIX A

### Metadata relating to each species identified in at least one of 14 *Apis mellifera* hives in northwest England. Information includes the species name, status (native, non‐native or neophyte), a habitat descriptor, and whether it is a species plausibly found within the UK

**Table:**

Species	Status	Habit	Habitat
*Impatiens glandulifera*	Neophyte	Annual	Manmade, moist soils
*Rubus armeniacus*	Non_native	Shrub	Manmade
*Olea europaea*	Non_native	Tree	Manmade
*Melilotus officinalis*	Neophyte	Biennial	Wide range
*Citrus sinensis*	Non_native	Tree	Manmade
*Viburnum acerifolium*	Neophyte	Shrub	Manmade
*Cannabis sativa*	Non_native	Annual	Manmade
*Salix babylonica*	Non_native	Tree	Manmade
*Trifolium repens*	Native	Perennial herb	Wide range
*Phormium tenax*	Non_native	Perennial	Manmade
*Zanthoxylum piperitum*	Non_native	Shrub	Manmade
*Papaver somniferum*	Non_native	Annual	Manmade
*Lavatera thuringiaca*	Non_native	Perennial herb	Manmade
*Actinidia chinensis*	Non_native	Vine	Manmade
*Ailanthus altissima*	Neophyte	Tree	Manmade
*Papaver rhoeas*	Native	Annual	Manmade, disturbed habitats
*Leontodon hispidus*	Native	Perennial herb	Wide range, calcareous soils
*Ligustrum ovalifolium*	Neophyte	Shrub	Manmade
*Filipendula ulmaria*	Native	Perennial herb	Wetland
*Prunus avium*	Native	Tree	Wide range
*Phaseolus coccineus*	Non_native	Vine	Manmade
*Sinapis arvensis*	Native	Annual	Disturbed habitats
*Castanea sativa*	Neophyte	Tree	Manmade, woodland
*Melilotus altissimus*	Neophyte	Biennial	Disturbed habitats

## APPENDIX B

### List of sources from which each species status was identified. Sources are either online databases, books of native flora, or specialist websites devoted to a taxon

**Table:**

Species	Status_source
*Actinidia chinensis*	https://pfaf.org/USER/Plant.aspx?LatinName=Actinidia+chinensis
*Ailanthus altissima*	https://pfaf.org/user/Plant.aspx?LatinName=Ailanthus+altissima
*Cannabis sativa*	https://pfaf.org/user/Plant.aspx?LatinName=Cannabis+sativa
*Castanea sativa*	Stace, C. *New Flora of the British Isles* (3rd ed.)
*Citrus sinensis*	https://pfaf.org/user/Plant.aspx?LatinName=Citrus+sinensis
*Filipendula ulmaria*	https://pfaf.org/user/Plant.aspx?LatinName=Filipendula+ulmaria
*Impatiens glandulifera*	Stace, C. *New Flora of the British Isles* (3rd ed.)
*Lavatera thuringiaca*	https://pfaf.org/User/Plant.aspx?LatinName=Lavatera+thuringiaca
*Leontodon hispidus*	https://pfaf.org/user/plant.aspx?LatinName=Leontodon+hispidus
*Ligustrum ovalifolium*	Stace, C. *New Flora of the British Isles* (3rd ed.)
*Melilotus altissimus*	Stace, C. *New Flora of the British Isles* (3rd ed.)
*Melilotus officinalis*	https://pfaf.org/user/plant.aspx?LatinName=Melilotus+officinalis
*Olea europaea*	https://pfaf.org/user/Plant.aspx?LatinName=Olea+europaea
*Papaver rhoeas*	https://pfaf.org/user/Plant.aspx?LatinName=Papaver+rhoeas
*Papaver somniferum*	https://pfaf.org/user/plant.aspx?LatinName=Papaver+somniferum
*Phaseolus coccineus*	https://pfaf.org/user/plant.aspx?LatinName=Phaseolus+coccineus
*Phormium tenax*	https://pfaf.org/user/plant.aspx?latinname=Phormium+tenax
*Prunus avium*	Stace, C. *New Flora of the British Isles* (3rd ed.)
*Rubus armeniacus*	Allen, D. E. (2003). *Rubus in Surrey* (p. 18). Surrey Botanical Society
*Salix babylonica*	Stace, C. *New Flora of the British Isles* (3rd ed.)
*Sinapis arvensis*	https://pfaf.org/user/Plant.aspx?LatinName=Sinapis+arvensis
*Trifolium repens*	https://pfaf.org/user/plant.aspx?LatinName=Trifolium+repens
*Viburnum acerifolium*	https://www.classicviburnums.com/index.cfm/fuseaction/plants.plantDetail/plant_id/7033/index.htm
*Zanthoxylum piperitum*	https://pfaf.org/user/plant.aspx?latinname=Zanthoxylum+piperitum

## APPENDIX C

### List of sources from which each species' habit was identified. Sources are either online databases, books of native flora, or specialist websites devoted to a taxon

**Table:**

Species	Habit_source
*Actinidia chinensis*	https://pfaf.org/USER/Plant.aspx?LatinName=Actinidia+chinensis
*Ailanthus altissima*	https://pfaf.org/user/Plant.aspx?LatinName=Ailanthus+altissima
*Cannabis sativa*	https://pfaf.org/user/Plant.aspx?LatinName=Cannabis+sativa
*Castanea sativa*	Stace, C. *New Flora of the British Isles* (3rd ed.)
*Citrus sinensis*	https://pfaf.org/user/Plant.aspx?LatinName=Citrus+sinensis
*Filipendula ulmaria*	https://pfaf.org/user/Plant.aspx?LatinName=Filipendula+ulmaria
*Impatiens glandulifera*	Stace, C. *New Flora of the British Isles*
*Lavatera thuringiaca*	https://pfaf.org/User/Plant.aspx?LatinName=Lavatera+thuringiaca
*Leontodon hispidus*	https://pfaf.org/user/plant.aspx?LatinName=Leontodon+hispidus
*Ligustrum ovalifolium*	https://pfaf.org/user/plant.aspx?latinname=Ligustrum+ovalifolium
*Melilotus altissimus*	https://pfaf.org/user/Plant.aspx?LatinName=Melilotus+altissimus
*Melilotus officinalis*	https://pfaf.org/user/plant.aspx?LatinName=Melilotus+officinalis
*Olea europaea*	https://pfaf.org/user/Plant.aspx?LatinName=Olea+europaea
*Papaver rhoeas*	https://pfaf.org/user/Plant.aspx?LatinName=Papaver+rhoeas
*Papaver somniferum*	https://pfaf.org/user/plant.aspx?LatinName=Papaver+somniferum
*Phaseolus coccineus*	https://pfaf.org/user/plant.aspx?LatinName=Phaseolus+coccineus
*Phormium tenax*	https://pfaf.org/user/plant.aspx?latinname=Phormium+tenax
*Prunus avium*	https://pfaf.org/user/plant.aspx?latinname=Prunus+avium
*Rubus armeniacus*	http://www.surreyflora.org.uk/Documents/flora05.pdf
*Salix babylonica*	https://pfaf.org/User/Plant.aspx?LatinName=Salix+babylonica
*Sinapis arvensis*	https://pfaf.org/user/Plant.aspx?LatinName=Sinapis+arvensis
*Trifolium repens*	https://pfaf.org/user/plant.aspx?LatinName=Trifolium+repens
*Viburnum acerifolium*	https://www.classicviburnums.com/index.cfm/fuseaction/plants.plantDetail
*Zanthoxylum piperitum*	https://pfaf.org/user/plant.aspx?latinname=Zanthoxylum+piperitum

## APPENDIX D

### *rbcL* read counts attributed to taxa plausibly detected in the UK assigned genus or species, per apiary

**Table:**

Order	Family	Genus	Species	A	B	C	D	E	F	G	H	I	J	K	L	M	N
Ericales	Balsaminaceae	*Impatiens*	*Impatiens glandulifera*	0	3597	13,982	245	18,419	0	6005	8142	0	1002	117,419	78,123	47,140	83,885
Rosales	Rosaceae	*Rubus*	*Rubus armeniacus*	35,405	98,362	65,554	4433	0	24,856	6538	8068	0	0	0	0	0	0
Lamiales	Oleaceae	*Olea*	*Olea europaea*	10,807	4265	10,164	8737	8714	0	5561	34,799	0	60,948	0	0	0	0
Fabales	Fabaceae	*Melilotus*	*Melilotus officinalis*	0	0	7149	0	0	68,779	0	0	0	0	0	0	0	0
Sapindales	Rutaceae	*Citrus*	*Citrus sinensis*	0	0	0	0	0	0	0	0	63,728	0	0	0	0	0
Dipsacales	Adoxaceae	*Viburnum*	*Viburnum acerifolium*	0	0	0	0	0	0	0	0	0	20,690	0	0	0	0
Rosales	Cannabaceae	*Cannabis*	*Cannabis sativa*	0	0	0	0	0	0	0	0	0	0	12,107	0	0	0
Malpighiales	Salicaceae	*Salix*	*Salix babylonica*	0	0	0	0	0	0	5963	1269	0	0	0	0	0	0
Fabales	Fabaceae	*Trifolium*	*Trifolium repens*	0	1323	0	0	0	0	1391	0	0	910	0	0	0	0
Asparagales	Xanthorrhoeaceae	*Phormium*	*Phormium tenax*	3513	0	0	0	0	0	0	0	0	0	0	0	0	0
Sapindales	Rutaceae	*Zanthoxylum*	*Zanthoxylum piperitum*	0	0	0	2396	0	0	0	0	0	0	0	0	0	0
Ranunculales	Papaveraceae	*Papaver*	*Papaver somniferum*	0	0	0	0	0	0	0	1139	0	0	0	0	0	0
Malvales	Malvaceae	*Lavatera*	*Lavatera thuringiaca*	0	0	0	452	0	0	0	0	0	0	0	0	0	0
Ericales	Actinidiaceae	*Actinidia*	*Actinidia chinensis*	0	0	0	0	0	0	0	594	0	0	0	0	0	0
Sapindales	Simaroubaceae	*Ailanthus*	*Ailanthus altissima*	0	0	0	176	0	0	0	0	0	0	0	0	0	0
Ranunculales	Papaveraceae	*Papaver*	*Papaver rhoeas*	0	0	0	193	0	0	0	0	0	0	0	0	0	0
Rosales	Rosaceae	*Rubus*	NA	17,270	64,271	3559	1225	0	14,038	14,021	10,975	0	0	0	0	0	20,439
Rosales	Rosaceae	*Sorbus*	NA	26,708	0	92,585	0	13,587	0	0	0	0	0	0	0	0	0
Fagales	Fagaceae	*Quercus*	NA	1758	0	20,225	0	0	5569	3724	0	0	0	19,054	0	0	11,249
Sapindales	Rutaceae	*Citrus*	NA	0	0	0	0	0	0	0	0	534	0	0	0	0	0
Apiales	Araliaceae	*Hydrangea*	NA	0	0	0	0	13,049	0	0	671	0	46,135	0	0	0	0
Rosales	Rosaceae	*Prunus*	NA	11,186	1628	0	17,350	0	0	0	12,576	0	0	0	0	0	0
Ericales	Theaceae	*Camellia*	NA	0	0	0	0	0	0	27,499	0	0	0	1895	0	0	0
Rosales	Rosaceae	*Crataegus*	NA	0	0	0	0	22,495	0	0	680	0	0	0	0	0	0
Ericales	Ericaceae	*Rhododendron*	NA	0	0	0	0	0	0	0	0	0	0	8008	20,585	0	0
Rosales	Rosaceae	*Mespilus*	NA	2209	16,971	0	0	0	0	0	0	0	0	0	0	0	0
Rosales	Rosaceae	*Fragaria*	NA	0	0	8106	0	0	0	0	9882	0	0	0	0	0	0
Lamiales	Scrophulariaceae	*Buddleja*	NA	2632	14,726	0	664	0	0	0	592	0	0	0	0	0	0
Rosales	Rosaceae	*Malus*	NA	6547	0	0	410	0	0	0	1714	655	0	0	0	0	0
Malvales	Malvaceae	*Tilia*	NA	0	7094	0	355	3230	0	0	940	0	0	0	0	0	0
Dipsacales	Caprifoliaceae	*Dipsacus*	NA	0	0	0	0	0	0	0	0	0	0	0	0	0	9852
Malpighiales	Salicaceae	*Salix*	NA	0	0	0	1866	0	0	0	4128	0	0	0	0	0	0
Sapindales	Sapindaceae	*Acer*	NA	4865	1659	0	0	0	0	0	0	0	0	0	0	0	0
Cucurbitales	Begoniaceae	*Begonia*	NA	0	0	0	0	0	0	7052	0	0	0	4078	0	0	0
Rosales	Rosaceae	*Pyrus*	NA	0	0	0	2545	0	0	0	1172	0	0	0	0	0	0
Myrtales	Myrtaceae	*Eucalyptus*	NA	0	0	0	0	0	0	0	0	0	0	2847	0	0	0
Rosales	Rosaceae	*Rosa*	NA	1869	0	0	0	0	0	0	0	0	0	0	0	0	0
Fagales	Juglandaceae	*Juglans*	NA	0	0	0	0	0	0	0	0	1659	0	0	0	0	0
Lamiales	Lamiaceae	*Salvia*	NA	0	1494	0	0	0	0	0	0	0	0	0	0	0	0
Geraniales	Geraniaceae	*Geranium*	NA	0	0	0	0	0	0	0	1407	0	0	0	0	0	0
Aquifoliales	Aquifoliaceae	*Ilex*	NA	0	0	0	0	0	0	0	0	0	0	1014	0	0	0
Rosales	Rhamnaceae	*Ceanothus*	NA	0	0	0	0	0	0	0	741	0	0	0	0	0	0
Magnoliales	Magnoliaceae	*Magnolia*	NA	0	0	0	0	0	0	0	0	0	0	401	0	0	0
Solanales	Convolvulaceae	*Calystegia*	NA	0	0	0	0	0	0	0	627	0	0	0	0	0	0
Poales	Poaceae	*Triticum*	NA	0	0	0	0	0	0	0	0	58	0	0	0	0	0
Solanales	Solanaceae	*Nicotiana*	NA	0	0	0	0	0	0	0	0	33	0	0	0	0	0

## APPENDIX E

### ITS2p read counts attributed to taxa plausibly detected in the UK assigned genus or species, per apiary

**Table:**

Order	Family	Genus	Species	A	B	C	D	E	F	G	H	I	J	K	L	M	N
Ericales	Balsaminaceae	*Impatiens*	*Impatiens glandulifera*	35,988	122,419	195,927	41,772	124,400	105,386	209,250	212,860	1897	300,533	290,665	163,508	151,696	165,628
Fabales	Fabaceae	*Trifolium*	*Trifolium repens*	4244	1848	0	888	0	1166	0	0	0	0	0	0	0	0
Asterales	Asteraceae	*Leontodon*	*Leontodon hispidus*	0	0	0	0	0	0	0	9631	0	0	0	0	0	0
Lamiales	Oleaceae	*Ligustrum*	*Ligustrum ovalifolium*	0	0	0	24,897	0	0	6968	7105	0	0	0	0	0	0
Rosales	Rosaceae	*Filipendula*	*Filipendula ulmaria*	0	0	0	0	0	0	0	17,128	0	0	0	0	0	0
Rosales	Rosaceae	*Prunus*	*Prunus avium*	0	0	0	11,109	0	0	0	0	0	0	0	0	0	0
Fabales	Fabaceae	*Phaseolus*	*Phaseolus coccineus*	0	0	0	0	0	0	19,337	0	0	0	0	0	0	0
Brassicales	Brassicaceae	*Sinapis*	*Sinapis arvensis*	3634	2038	3908	1561	0	0	0	0	0	0	0	0	0	0
Fagales	Fagaceae	*Castanea*	*Castanea sativa*	0	0	0	0	2014	0	0	0	1114	0	0	0	0	0
Fabales	Fabaceae	*Melilotus*	*Melilotus altissimus*	7230	0	0	1664	0	0	0	0	0	0	0	0	0	0
Ericales	Balsaminaceae	*Impatiens*	NA	19,606	74,375	121,599	24,606	94,358	66,455	138,572	119,756	3455	179,160	181,049	80,596	111,081	87,524
Rosales	Rosaceae	*Rubus*	NA	21,719	107,815	3492	77,654	8417	1332	8075	22,460	0	0	0	0	1275	2501
Fabales	Fabaceae	*Melilotus*	NA	4592	0	1297	0	0	0	0	0	0	0	0	0	0	0
Sapindales	Rutaceae	*Citrus*	NA	0	0	0	0	0	0	0	0	123,240	0	0	0	0	0
Apiales	Araliaceae	*Hydrangea*	NA	0	0	0	0	3816	0	0	0	0	0	0	0	0	0
Rosales	Rosaceae	*Prunus*	NA	1084	0	0	19,995	0	0	0	2619	0	0	0	0	0	0
Malpighiales	Salicaceae	*Salix*	NA	547	0	0	17,756	0	0	0	1641	0	0	0	0	0	0
Fabales	Fabaceae	*Trifolium*	NA	2825	0	0	0	0	0	0	0	0	0	0	0	0	0
Fagales	Juglandaceae	*Juglans*	NA	0	0	0	0	0	0	0	0	22,445	0	0	0	0	0
Malpighiales	Salicaceae	*Populus*	NA	0	0	0	0	0	0	0	0	0	0	0	0	0	43,523
Fabales	Fabaceae	*Phaseolus*	NA	0	0	0	0	0	0	32,027	0	0	0	0	0	0	0
Fabales	Fabaceae	*Caragana*	NA	3364	0	0	0	0	2089	1885	0	0	0	0	0	0	0

## APPENDIX F

### Total numbers of genus and species detected by each marker (*rbcL* or ITS2p) in each of 14 *Apis mellifera* hives in north west England

**Table:**

Hive	rbcL Genera	rbcL Species	ITS2p Genera	ITS2p Species
A	11	3	8	4
B	10	4	4	3
C	7	4	4	2
D	13	7	8	6
E	6	2	4	2
F	3	2	4	2
G	8	5	5	3
H	17	6	7	4
I	5	1	4	2
J	5	4	1	1
K	9	2	1	1
L	2	1	1	1
M	1	1	2	1
N	4	1	3	1
